# Comprehensive molecular analyses of an autoimmune-related gene predictive model and immune infiltrations using machine learning methods in moyamoya disease

**DOI:** 10.3389/fmolb.2022.991425

**Published:** 2022-12-20

**Authors:** Shifu Li, Ying Han, Qian Zhang, Dong Tang, Jian Li, Ling Weng

**Affiliations:** ^1^ Department of Neurosurgery, Xiangya Hospital, Central South University, Changsha, Hunan, China; ^2^ National Clinical Research Center for Geriatric Disorders, Central South University, Changsha, Hunan, China; ^3^ Department of Oral and Maxillofacial Surgery, Center of Stomatology, Xiangya Hospital of Central South University, Changsha, Hunan, China; ^4^ Center for Medical Genetics and Hunan Key Laboratory of Medical Genetics, School of Life Sciences, Central South University, Changsha, Hunan, China; ^5^ Hydrocephalus Center, Xiangya Hospital, Central South University, Changsha, Hunan, China; ^6^ Department of Neurology, Xiangya Hospital, Central South University, Changsha, Hunan, China; ^7^ Engineering Research Center of Hunan Province in Cognitive Impairment Disorders, Central South University, Changsha, China; ^8^ Hunan International Scientific and Technological Cooperation Base of Neurodegenerative and Neurogenetic Diseases, Changsha, China; ^9^ Key Laboratory of Hunan Province in Neurodegenerative Disorders, Central South University, Changsha, China

**Keywords:** moyamoya disease, machine learning, bioinformatics, immune infiltration, autoimmune-related genes

## Abstract

**Background:** Growing evidence suggests the links between moyamoya disease (MMD) and autoimmune diseases. However, the molecular mechanism from genetic perspective remains unclear. This study aims to clarify the potential roles of autoimmune-related genes (ARGs) in the pathogenesis of MMD.

**Methods:** Two transcription profiles (GSE157628 and GSE141025) of MMD were downloaded from GEO databases. ARGs were obtained from the Gene and Autoimmune Disease Association Database (GAAD) and DisGeNET databases. Differentially expressed ARGs (DEARGs) were identified using “limma” R packages. GO, KEGG, GSVA, and GSEA analyses were conducted to elucidate the underlying molecular function. There machine learning methods (LASSO logistic regression, random forest (RF), support vector machine-recursive feature elimination (SVM-RFE)) were used to screen out important genes. An artificial neural network was applied to construct an autoimmune-related signature predictive model of MMD. The immune characteristics, including immune cell infiltration, immune responses, and HLA gene expression in MMD, were explored using ssGSEA. The miRNA-gene regulatory network and the potential therapeutic drugs for hub genes were predicted.

**Results:** A total of 260 DEARGs were identified in GSE157628 dataset. These genes were involved in immune-related pathways, infectious diseases, and autoimmune diseases. We identified six diagnostic genes by overlapping the three machine learning algorithms: CD38, PTPN11, NOTCH1, TLR7, KAT2B, and ISG15. A predictive neural network model was constructed based on the six genes and presented with great diagnostic ability with area under the curve (AUC) = 1 in the GSE157628 dataset and further validated by GSE141025 dataset. Immune infiltration analysis showed that the abundance of eosinophils, natural killer T (NKT) cells, Th2 cells were significant different between MMD and controls. The expression levels of HLA-A, HLA-B, HLA-C, HLA-DMA, HLA-DRB6, HLA-F, and HLA-G were significantly upregulated in MMD. Four miRNAs (mir-26a-5p, mir-1343-3p, mir-129-2-3p, and mir-124-3p) were identified because of their interaction at least with four hub DEARGs.

**Conclusion:** Machine learning was used to develop a reliable predictive model for the diagnosis of MMD based on ARGs. The uncovered immune infiltration and gene-miRNA and gene-drugs regulatory network may provide new insight into the pathogenesis and treatment of MMD.

## Introduction

Moyamoya disease (MMD) is an uncommon, chronic cerebrovascular disorder characterized by progressive occlusion of the supraclinoid internal carotid artery (ICA) and its main branches within the circle of Willis. MMD, also known as an abnormal netlike vascular disease at the base of the brain, is a term coined by Suzuki and Takaku in 1969 to describe the classic angiographic appearance—a puff of cigarette smoke drifting in the air (Suzuki and Takaku, 1969). Important clinical features include ischemic stroke, often presented in childhood, and hemorrhagic stroke, generally observed in adults. The incidence rate is twice higher in females than males, and children around 5 years and adults in their mid-40s are particularly affected (Kuroda and Houkin, 2008). MMD is the most common pediatric cerebrovascular disease in Japan, affecting approximately three out of every 100,000 children (Scott and Smith, 2009). MMD conventionally refers to patients with above idiopathic pathology without a previously diagnosed condition. Distinct from the definitive MMD, Moyamoya (MM) syndrome (also named Quasi-Moyamoya disease, secondary Moyamoya disease, and akin-Moyamoya disease) is the occurrence of angiographic MM in association with acquired (i.e., autoimmune diseases) or inherited disorders [i.e., neurofibromatosis type 1, sickle cell anemia, Down syndrome (DS)] (Scott and Smith, 2009).

The exact etiology of MMD remains unknown; however, hereditary, immunogenic inflammatory, and hemodynamic factors are known to be responsible. The close relationship between patients with MM syndrome and autoimmune diseases, such as type 1 diabetes mellitus, thyroid disease, systemic lupus erythematosus (SLE), and DS has been reported (Huang et al., 2017). A study in a primarily white, midwestern United States population showed that the prevalence of autoimmune diseases was significantly higher in patients with MMD, particularly type 1 diabetes mellitus (8.5% *versus* 0.4% in the general population), thyroid disease (17.0% *versus* 8.0% in the institutional general patient population), and hyperlipidemia (27.7% *versus* 16.3% in the general population). A meta-analysis conducted in 2014 revealed that elevated thyroid autoantibodies and elevated thyroid function are independently associated risk factors for MMD (Lei et al., 2014). Autoimmunity is the main link between SLE and MM syndrome since immune complexes lead to vasculitis and narrow or occluded vessels (El Ramahi and Al Rayes, 2000; Jeong et al., 2008). Chen et al. (2016) found that the overall prevalence of autoimmune diseases in patients with unilateral MMD was significantly higher than that in patients with bilateral MMD. Although the close relationships between autoimmune diseases and MDD diseases have been recognized, the underlying mechanisms remain to be clarified.

Advances in molecular biology and next-generation sequencing technologies have made it possible to study disease mechanisms at the genetic and mRNA levels. Gene expression profiling through methods such as microarray and RNA sequencing based on the Gene Expression Omnibus (GEO) database is widely used to explore differentially expressed genes (DEGs), analyze potential function pathways, and determine molecular mechanisms involved in various cerebrovascular diseases (Chen et al., 2022). A recent bioinformatics study identified the potential neutrophil-associated genes in MMD (Jin and Duan, 2022). However, the role of autoimmune-related genes (ARGs) in the pathophysiology of MMD is still unclear. In recent years, the development of machine learning algorithms has provided more choices for diagnostic models as precision medical predictive tools. Our study integrated least absolute shrinkage and selection operator (LASSO) logistic regression, random forest (RF), support vector machine-recursive feature elimination (SVM-RFE), and artificial neural network to screen and identify diagnostic markers and construct an autoimmune-related signature predictive model of MMD. The immune characteristics, including immune cell infiltration, immune responses, and HLA gene expression in MMD, were explored. The miRNA-gene regulatory network and the potential therapeutic drugs for hub genes were predicted.

## Materials and methods

### Downloading and processing of data

Microarray data containing two transcription profiles (GSE157628 and GSE141025) were downloaded from the NCBI GEO database (https://www.ncbi.nlm.nih.gov/geo/). The dataset of GSE157628 was utilized as the exploratory dataset, and the GSE141025 profile acted as the validation dataset. The GSE157628 profile included micro-samples of the middle cerebral artery (MCA) collected from 11 patients with MMD and nine age- and gender-matched control samples (three from patients with epilepsy and six from patients with ICA aneurysms) at the platform of GPL16699. The expression profiles of MCA samples from four patients with MMD and four matched superficial temporal artery controls in GSE141025 were extracted for dataset validation. If multiple probes matched one gene, the probe with the maximal median expression values was annotated into the homologous gene symbol through the platform’s annotation information.

### Collection of autoimmune-related genes

ARGs were obtained from the Gene and Autoimmune Disease Association Database (GAAD) (Lu et al., 2018) and DisGeNET databases (Piñero et al., 2020) after deleting duplicate genes. GAAD contained 44,762 associations between 49 autoimmune-related diseases and 4,249 genes through text mining and manual curation. DisGeNET (v7.0), one of the largest publicly available collections of genes and variants associated with human diseases, contained 1,134,942 gene-disease associations (GDAs), between 21,671 genes and 30,170 diseases, disorders, traits, and clinical or abnormal human phenotypes.

### Identification of differentially expressed autoimmune-related genes

The principal component analysis was conducted by using the factoextra R package. To identify the DEARGs, we performed differential expression analysis using the “limma” package in R software to detect DEGs between the MMD and control groups in the training dataset (GSE157628). The DEGs were screened with the criteria of |log2FoldChange| > 1 and *p* < 0.05. Volcano maps and clustering heatmaps were prepared to visualize the differences using the “ggplot2” and “ComplexHeatmap” packages in the R software. We intersected the DEGs with ARGs to identify DEARGs and visualized them with the “VennDiagram” package.

### Functional enrichments between moyamoya disease tissues and controls

To uncover the biological function in MMD, gene set enrichment analysis (GSEA) was used to enrich the Kyoto Encyclopedia of Genes and Genomes (KEGG) pathways using the “clusterProfiler” package. Gene set variation analysis (GSVA) algorithm was used to calculate the Reactome (“c2.cp.reactome.v7.5.1.symbols” gene set from the Molecular Signatures Database) (Liberzon et al., 2015) processes score using the “GSVA” R package.

### Authentication of the organ/tissue-specific expressed DEARGs

To understand the tissue/organ-specific expression of these DEARGs, we analyzed the gene distribution in tissues using the online tool BioGPS (http://biogps.org/) (Wu et al., 2009). The following criteria had to be met (Wang et al., 2020): 1) the expression level of transcripts mapped to a single organ system was >10 times the median, and 2) the second-highest level was not more than one-third of the highest expression level.

### Protein-protein interaction network and functional annotation of DEARGs

The PPI network of DEARGs was prepared using the online tool STRING (https://string-db.org/) with a minimum required interaction score of 0.4. We downloaded the interaction information and visualized the PPI network using Cytoscape software (v3.8.2). ClueGO, a plugin app of Cytoscape for the function enrichment, was used to annotate the biological processes (BP) of Gene Ontology (GO) and KEGG pathways of this network and the genes participating in these terms. The R package “DOSE” was used to perform Disease Ontology (DO) analysis (Yu et al., 2015).

### Screening for crucial DEARGs and candidate signatures

First, we applied five methods (Closeness, Degree, MCC(Maximal Clique Centrality), MNC (Maximum neighborhood component), and Radiality) in cytoHubba to select the top 30 genes and intersected them through the Venn plot to find the common genes (Chin et al., 2014). Based on common genes, we constructed a co-expression network *via* GeneMANIA (http://www.genemania.org/) to identify internal associations (Warde-Farley et al., 2010).

Second, to further identify the crucial DEARGs and candidate signatures, three machine learning [LASSO logistic regression, RF, and support vector machine (SVM)] algorithms were adopted. The LASSO logistic regression model was used to select optimal variables using the penalty coefficient. RF is a machine learning algorithm with an ensemble of multiple decision trees that combines the knowledge generated by a collection of individual trees using randomness. The top 10 variables were selected as the most important features in the methods. SVM is a supervised machine learning technique widely utilized for classification and regression. To avoid over-fitting, the SVM-RFE requires training multiple classifiers on subsets of features of decreasing size to search for the best features. In the present study, we overlapped genes identified by the three methods and designated the communal gens as our candidate signatures for constructing the next diagnostic model.

### Construction of the artificial neural network diagnostic model

We constructed a back propagation artificial neural network model using the “neuralnet” package. The expression profiles of the above screened signatures in the GSE157628 dataset were extracted and normalized. The min-max method was selected, and the data were mapped in the range of zero to one before training the neural network. The number of neurons was between the input and output layer sizes, usually two-thirds of the input size. A single hidden layer with four nodes was used. We calculated the classification score of the obtained disease neural network model as follows:

Predicting score=∑gene expression*neural network weight

The diagnostic ability was evaluated through the receiver operating characteristic (ROC) curve and confusion matrix. Another external dataset, GSE141025, was used to validate this model.

### Immune characteristics of the moyamoya disease microenvironment

xCell Aran et al. (2017), a novel gene signature-based method to identify 64 immune and stromal cell types, was used to score the abundance of immune cells in MMD. Single sample gene set enrichment analysis (ssGSEA) was used to analyze the immune cells and activities between MMD and controls. We also compared the expression levels of HLA molecules between the two groups. The significantly different immune characteristics were depicted with boxplots and heatmaps.

The association of the crucial gene signatures with the scores of infiltrating immune cells, immune activities, and expression of HLA molecules was explored using Pearson’s correlation analysis in R software. The resulting associations were visualized as a heatmap prepared with the “ggplot2” package.

### Prediction of a miRNA-genes regulatory network and potential drugs

NetworkAnalyst is a user-friendly online tool to create PPI networks, cell-type or tissue-specific PPI networks, gene regulatory networks, gene co-expression networks, and networks for toxicogenomics and pharmacogenomics studies (Zhou et al., 2019). We used the NetworkAnalyst to predict the miRNA-genes regulatory network through the Tarbase database (Karagkouni et al., 2018).

The Drug-Gene Interaction Database (DGIdb) (http://www.dgidb.org/) is an online database to predict drug-gene interaction based on the data mined from DrugBank, PharmGKB, Chembl, Drug Target Commons, and TTD. The DGIdb was searched to make predictions on potential molecule-related drugs that interact with crucial DEARGs. Only the drugs with identified interaction types persisted, and the Drugs-Genes interactions were visualized through a Sankey diagram.

### Sample collection and real-time quantitative polymerase chain reaction

We recruited ten patients who were diagnosed with MMD in Xiangya Hospital of Central South University for this research between June 2022 and October 2022. A total of 10 healthy controls with gender and age matched were also selected.

The detailed procedures of RT-qPCR were described in our previous studies (Li et al., 2022a; Li et al., 2022b). Birefly, peripheral blood monocytes (PBMCs) were isolated from the blood samples of patients and health persons. We extracted the total RNA from the PBMCs, performed reverse transcription reactions, and then amplified the cDNA. The results were analyzed using the 2^−ΔΔCT^ method and expressed as ratio of the internal control, GAPDH. The primer sequences used for RT-qPCR are listed in Supplementary Table S1.

## Results

### Differential expression analysis

**FIGURE 1:**
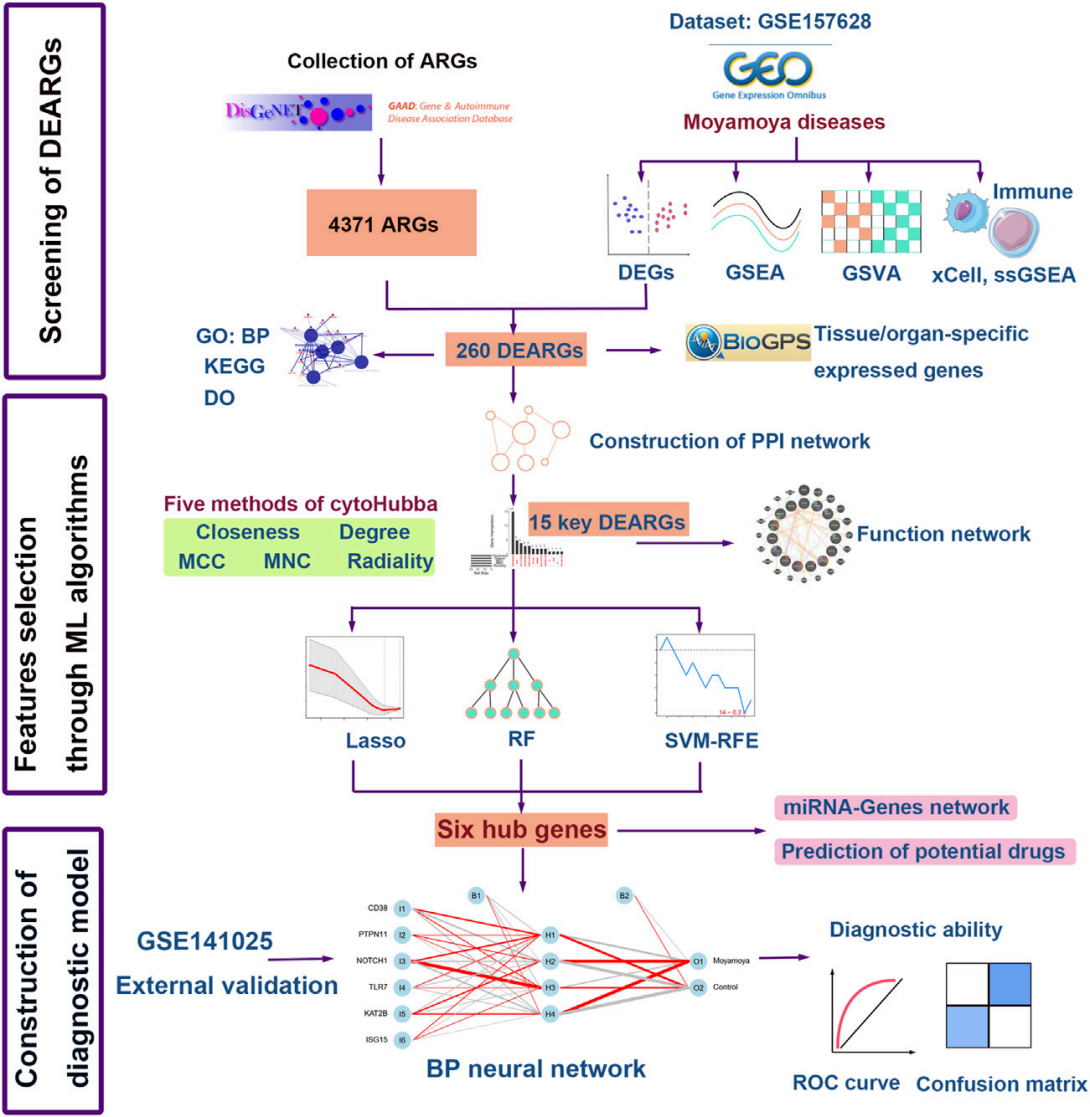
Flow chart of the study. DEGs, differentially expressed genes; GSEA, gene set enrichment analysis; ssGSEA, Single sample gene set enrichment; GSVA, gene set variation analysis; GO: BP, Gene Ontology: biological processes; KEGG, Kyoto Encyclopedia of Genes and Genomes; DO, Disease Ontology; ARGs, autoimmune-related genes; DEARGs, differentially expressed autoimmune-related genes; LASSO, the least absolute shrinkage and selection operation; RF, random forest; SVM-RFE, Support vector machine-recursive feature elimination; ML, machine learning; BP neural network, back propagation neural network; ROC, Receiver operating characteristic curve.

**FIGURE 2:**
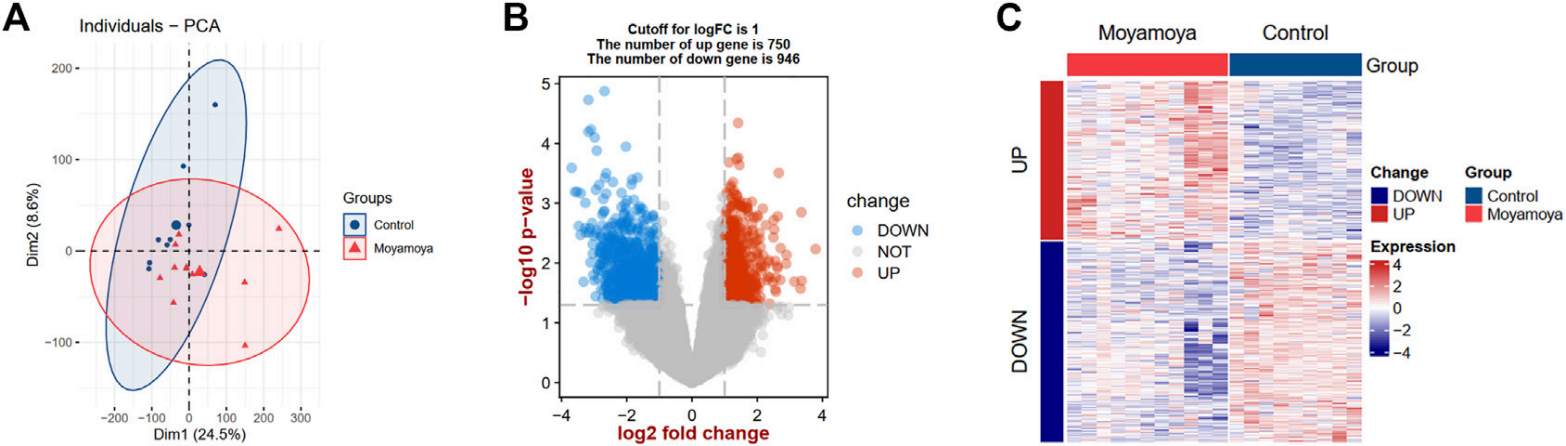
PCA and DEG analysis between MMD tissues and controls. **(A)** Principal component analysis between MMD tissues and controls. **(B)** A volcano plot shows the DEGs. Blue dots show the down-regulated genes and red dots represent the up-regulated genes. **(C)** A heat map shows the expression patterns of DEGs. PCA, principal component analysis; DEGs, differentially expressed genes; MMD, moyamoya disease.

The study flowchart is depicted in Figure 1. Principal component analysis showed that the MMD tissues and controls could be clearly distinguished in the GSE157628 dataset (Figure 2A). Differential expression analysis was further performed to screen for DEGs. Based on the selection criteria, 1696 DEGs (750 upregulated and 946 downregulated) were identified (Figure 2B). The expression patterns of these DEGs were visualized through a hierarchical clustering heatmap (Figure 2C).

### Functional enrichment analyses in moyamoya disease

**FIGURE 3:**
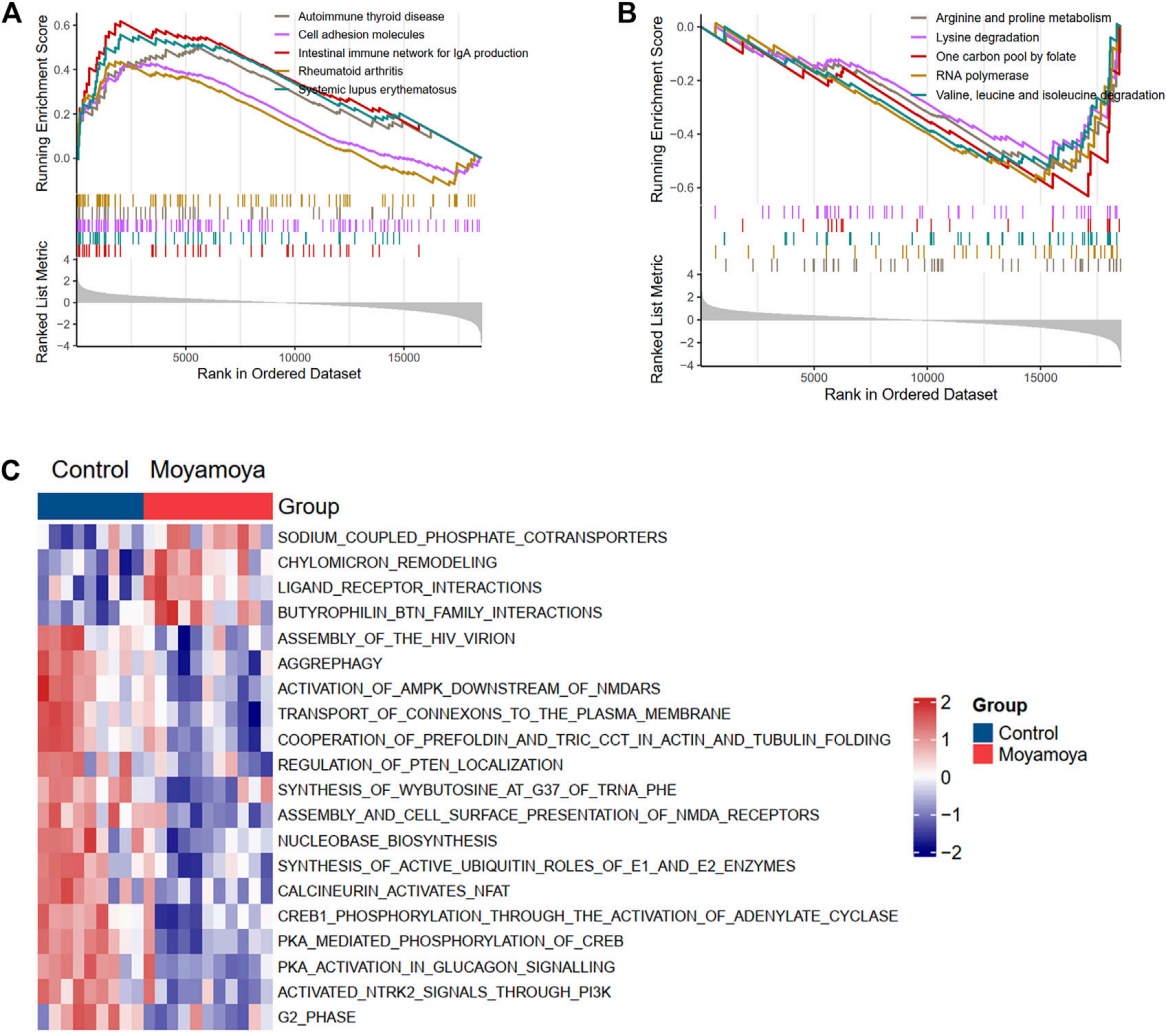
Biological KEGG and Reactome pathways involved in MMD based on GSEA and GSVA. **(A)** and **(B)** Up-regulated and down-regulated KEGG pathways from GSEA results, respectively. **(C)** The top differentially regulated reactome pathways from GSVA results.

GSEA and GSVA were performed to reveal the underlying biological pathways in MMD. The KEGG and Reactome analysis focus on the biological pathways. Compared with the comprehensive KEGG pathway, the Reactome pathway has more specific functions and focuses more on biochemical reactions. In our analysis, both methods were adopted. The KEGG analysis based on GSEA showed that the autoimmune thyroid disease, cell adhesion molecules, and rheumatoid arthritis pathways were upregulated (Figure 3A). In contrast, metabolism-related pathways (arginine and proline metabolism, lysine degradation, and one carbon pool by folate) were downregulated in MMD tissues (Figure 3B) compared with controls. The top 20 significantly differential Reactome pathways between MMD and controls are presented in Figure 3C, showing upregulated sodium-coupled phosphate cotransporters, chylomicron remodeling, and ligand-receptor interactions pathways. At the same time, aggrephagy and regulation of PTEN localization were downregulated in MMD tissues compared with controls (Figure 3C).

### Construction of protein-protein interaction network of DEARGs

**FIGURE 4:**
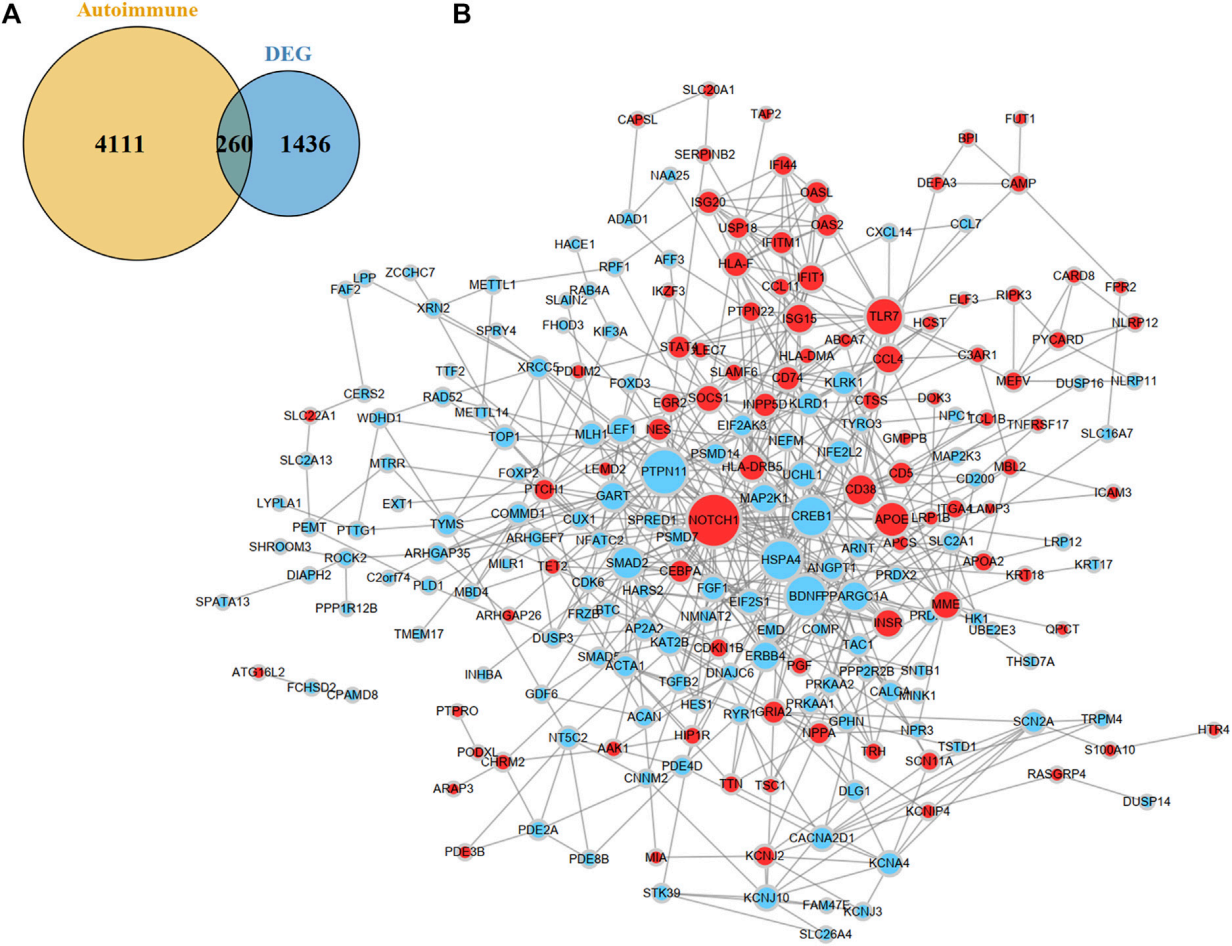
Identification and PPI network construction of DEARGs. **(A)** A venn plot show 260 DEARGs in MMD. **(B)** PPI network of DEARGs. The blue nodes represent the down-regulated genes and the red nodes represent the up-regulated genes. The dot size indicates the degree of the nodes. DEARGs, differentially expressed autoimmune-related genes.

After combining the GAAD and DisGeNET databases, 4371 ARGs were obtained. We overlapped the DEGs and ARGs, resulting in 260 DEARGs in MMD (Figure 4A). To elucidate the molecules’ functional associations, we imported these genes into the STRING database to construct a PPI network and further visualized it in Cytoscape. After removing nodes without interaction with other genes, a PPI network with 225 nodes and 602 edges was constructed (Figure 4B).

### Functional enrichment analyses of DEARGs

**FIGURE 5:**
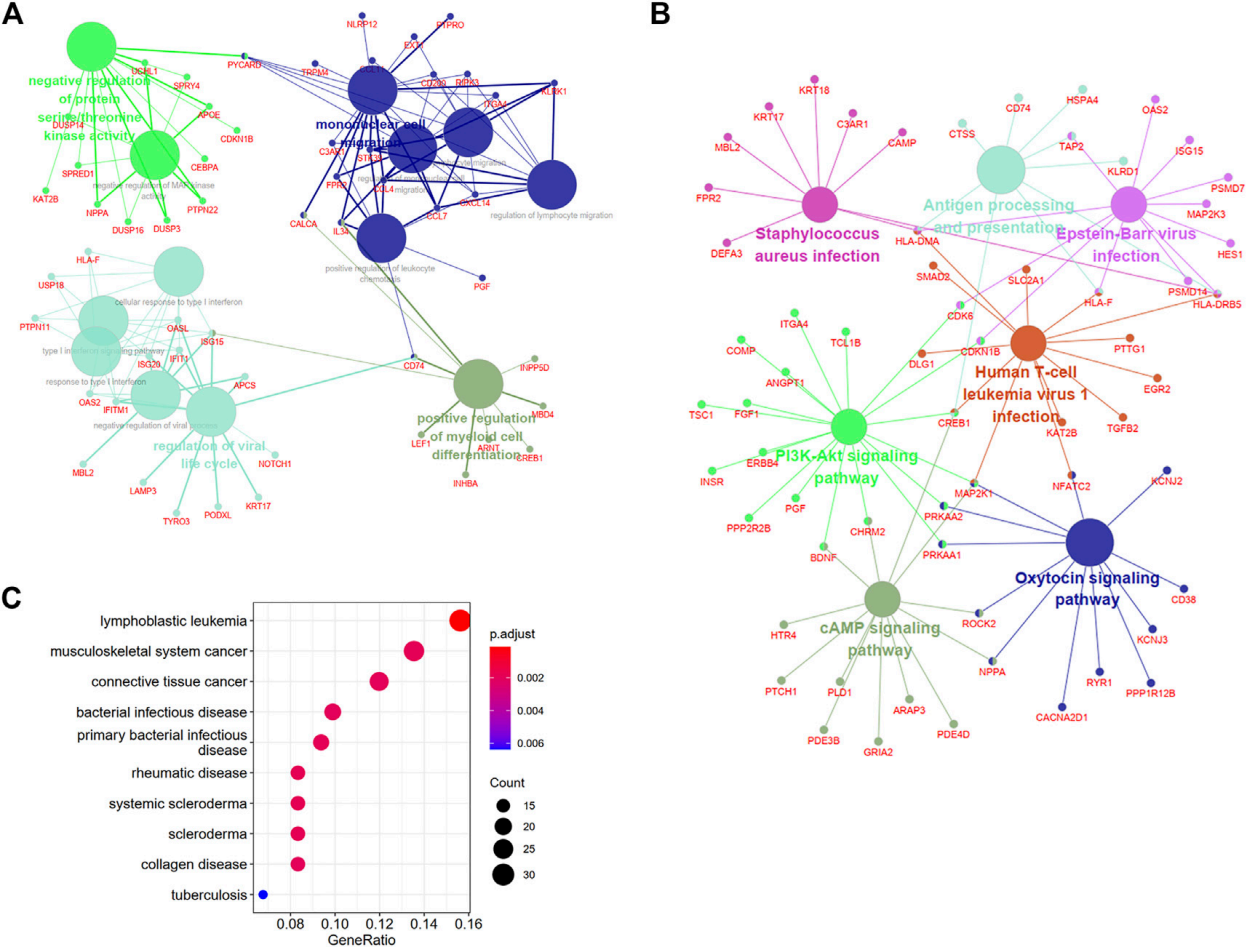
Biological annotations and DO analysis of DEARGs. **(A)** and **(B)** The enrichment network of biological processes and KEGG pathways with the participated genes in Cluego software. **(C)** The diseases of DEARGs involved through Disease Ontology analysis. DEARGs, differentially expressed autoimmune-related genes; DO, Disease Ontology.

For exploring the function and pathway of the DEARGs, we used ClueGO, a plugin of Cytoscape, and the “DOSE” package. The biological processes-genes network showed that mononuclear cell migration and regulation of viral life cycle were most enriched for DEARGs (Figure 5A and Supplementary Figure S1A). The cAMP signaling pathway, PI3K-Akt signaling pathway, antigen processing and presentation, and microbial infection-related pathways were enriched in the KEGG analysis (Figure 5B and Supplementary Figure S1B). To uncover the diseases that these genes may be involved in, a DO analysis was conducted, which showed that these genes participated in the development of different cancers, infectious diseases, and autoimmune diseases.

### Identification of tissue/organ-specific expressed genes

A total of 70 tissue/organ-specific expressed genes were identified for 260 DEARGs by BioGPS (Table 1). We observed that the system with the greatest distribution of tissue-specific expressed genes was the hematologic/immune system (30/70, 42.90%). The second organ-specific expressed system was the nervous system, which included 11 genes (11/70, 15.70%), followed by the digestive system (7/70, 10.00%), endocrine system (5/70, 7.10%), and genital system (4/70, 5.70%).

**TABLE 1:** 70 Identified tissue/organ-specific expressed genes by BioGPS.

System/Organ	Counts	Frequency (%)	Genes
Haematologic/Immune cells	30	42.90	TNFRSF17, CTSS, FPR2, HLA-DMA, HLA-DRB5, ICAM3, ITGA4, KLRD1, STAT4, MBD4, CARD8, KLRK1, PTPN22, TLR7, CCL4, INPP5D, NLRP12, PYCARD, OAS2, ISG20, KCNJ2, FCHSD2, FUT1, TOP1, CBLN2, XRN2, C3AR1, ZCCHC7, SPATA13, MILR1
Nervous	11	15.70	FGF1, GRIA2, PPP2R2B, TAC1, UCHL1, PPP1R17, SERPINI1, DNAJC6, PDE2A, AAK1, NMNAT2
Digestive	7	10.00	MBL2, SLC22A1, PEMT, CEBPA, SPRY4, APCS, APOA2
Endocrine	5	7.10	CALCA, CA3, FAM47E, SLC26A4, SPINK1
Genital	4	5.70	PGF, PRKAA1, DIAPH2, ARHGAP35
Bonemarrow	3	4.30	BPI, CAMP, DEFA3
Smooth muscle	3	4.30	BDNF, CCL7, CCL11
Adipocyte	2	2.90	COMP, MME
Immune organs	2	2.90	LEF1, NLRP11
Heart	1	1.40	NPPA
Respiratory	1	1.40	LAMP3
Tongue/Skeletal Muscle	1	1.40	TTN

### Screening for crucial DEARGs and candidate signatures

**FIGURE 6:**
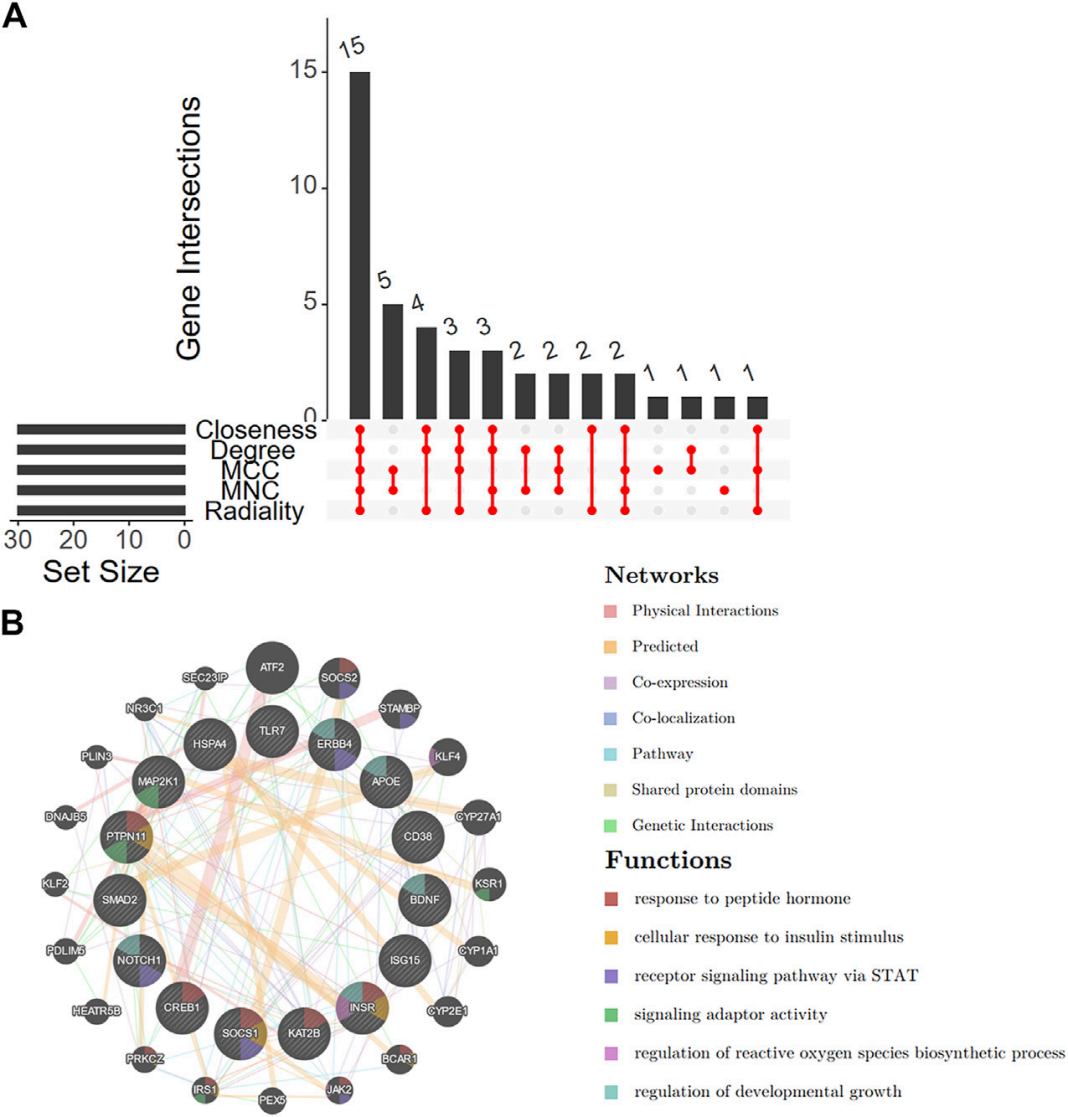
Venn diagram and co-expression of key DEARGs. **(A)** A Venn diagram shows that 15 key DEARGs are common genes from five cytoHubba methods. **(B)** 15 key DEARGs and their co-expressed genes analyzed by GENEMANIA. DEARGs, differentially expressed autoimmune-related genes.

First, we integrated five methods (Closeness, Degree, MCC, MNC, and Radiality) in cytoHubba and overlapped the top 30 genes from each method for robustness (Supplementary Figure S2 and Supplementary Table S2). A total of 15 shared genes were identified (Figure 6A). A co-expression network was constructed (Figure 6B), and 20 genes were identified that interacted with the 15 key DEARGs. In the complex PPI network, the interaction of the physical interactions accounted for 38.45%, predicted for 35.98%, co-expression for 20.16%, and colocalization for 3.44%.

**FIGURE 7:**
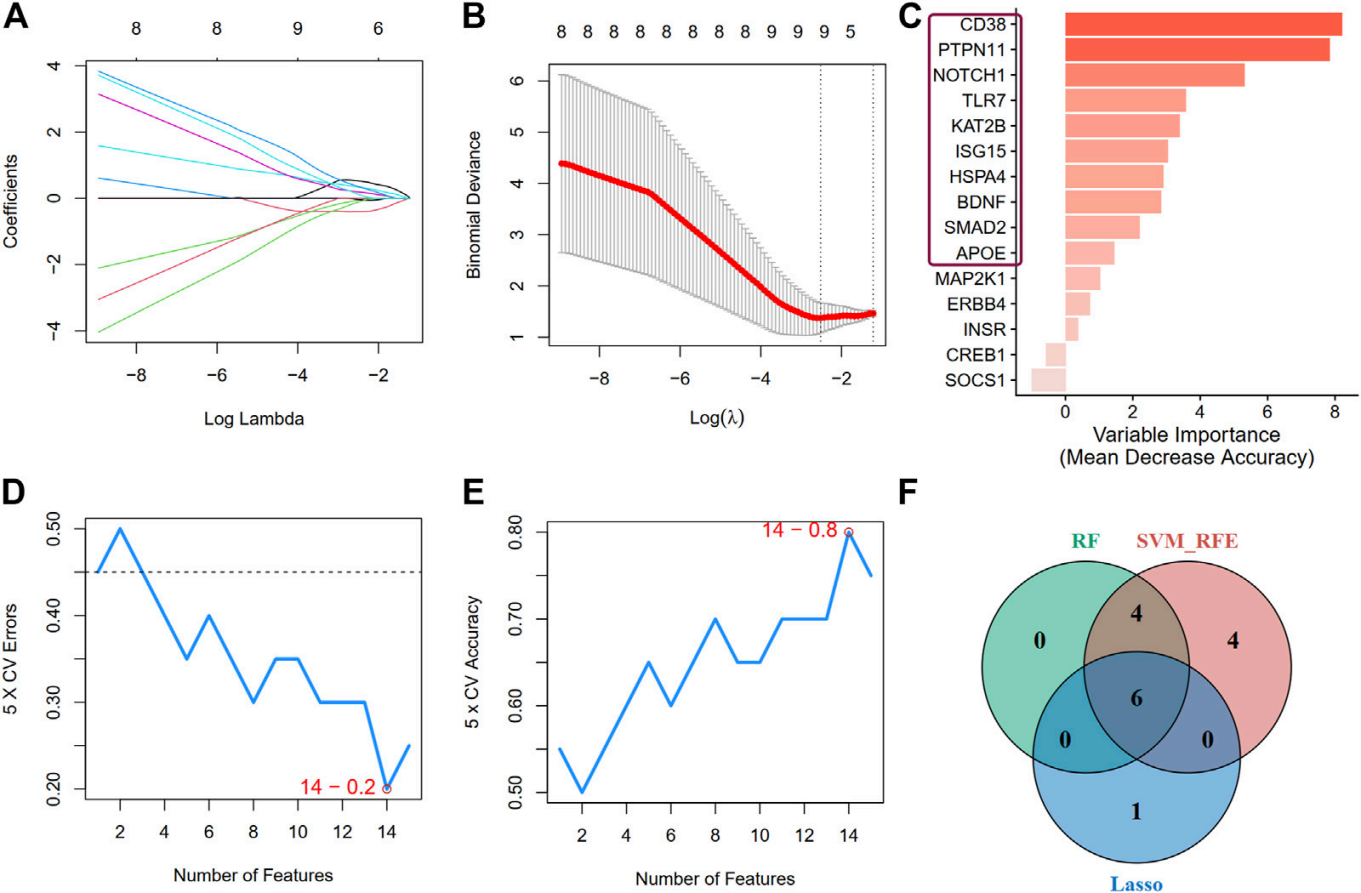
Screen for crucial DEARGs based on machine learning algorithms. **(A,B)** Feature selection by LASSO regression model **(A)** The coefficients change of different genes with different lambda **(B)** By verifying the optimal parameter (lambda) in the LASSO model, the partial likelihood deviance (binomial deviance) curve was plotted vs log(lambda). **(C)** The genes ranked by the feature importance based on random forest algorithm. The darker the color, the more important the gene is. **(D)** and **(E)** The error and accuracy of model changed with different number of features in support vector machine-recursive feature elimination method, respectively. **(F)** A Venn diagram demonstrating six diagnostic markers shared by the three algorithms.

Second, we applied three machine learning algorithms (LASSO regression, RF, and SVM-RFE) to screen further the most important signatures. Seven genes were determined by LASSO regression with the optimal values (Figures 7A, B). We selected the top 10 genes ranked by the variable importance in RF (Figure 7C). The error fell to the lowest perigee, and the accuracy reached the peak when the number of features was set to 14 in the SVM-RFE method (Figures 7D, E). We identified six diagnostic genes by overlapping the three algorithms (Figure 7F): *CD38*, *PTPN11*, *NOTCH1*, *TLR7*, *KAT2B*, and *ISG15*. The detailed descriptions of the six diagnostic signatures are listed in Table 2. The correlation among these genes was studied by Pearson’s method and presented with a heatmap (Supplementary Figure S3).

**TABLE 2:** Detail information about the six hub genes identified by machine learning.

Gene	Description	Chromosome	logFC	P.Value	Change
CD38	CD38 molecule	4	1.257	0.029	UP
PTPN11	protein tyrosine phosphatase non-receptor type 11	12	−1.021	0.007	DOWN
NOTCH1	notch receptor 1	9	1.104	0.005	UP
TLR7	toll like receptor 7	X	1.417	0.006	UP
KAT2B	lysine acetyltransferase 2B	3	−1.435	0.046	DOWN
ISG15	ISG15 ubiquitin like modifier	1	1.072	0.023	UP

### Construction and validation of the biological processes neural network diagnostic model

**FIGURE 8:**
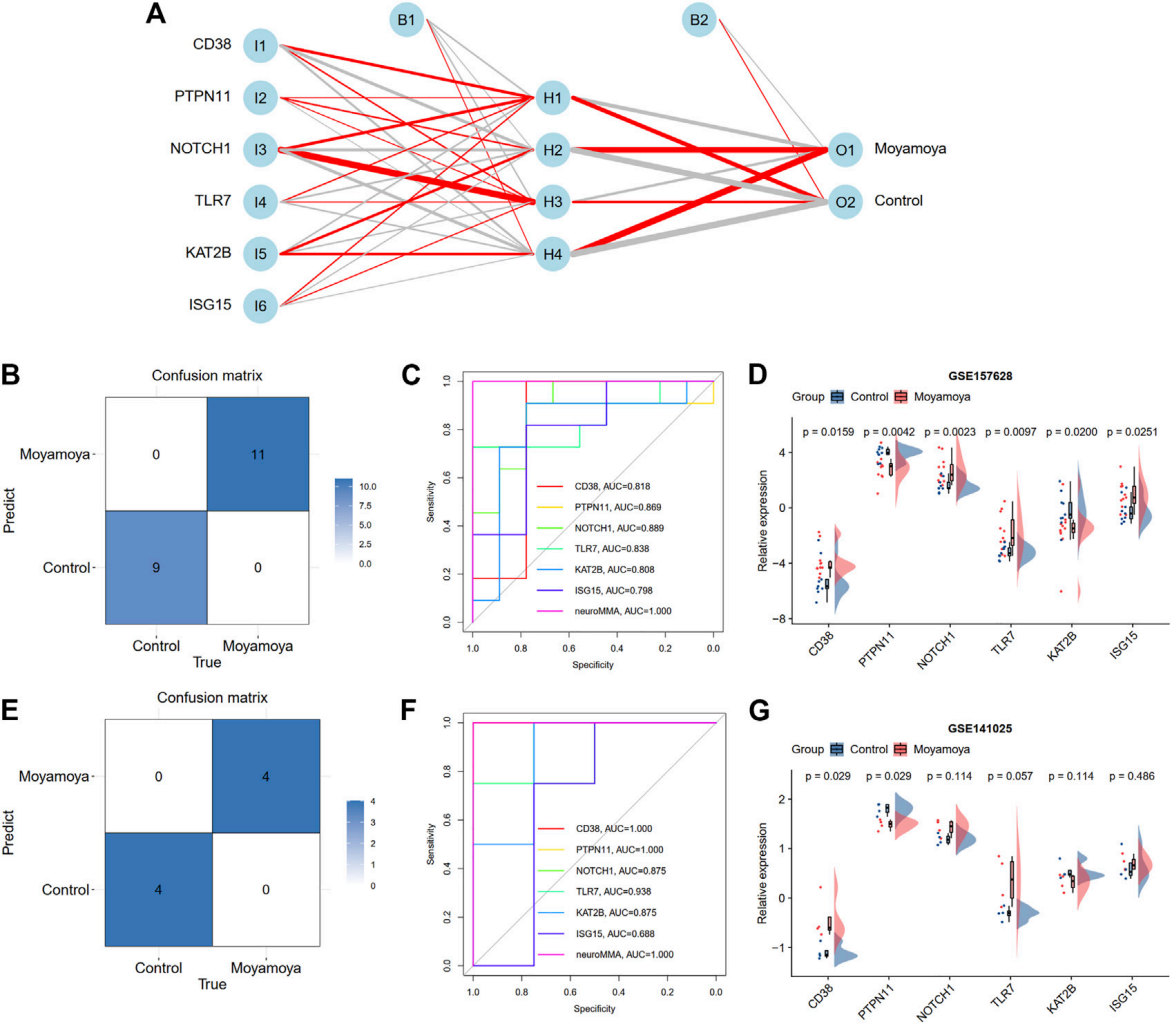
Construction and validation a BP neural network diagnostic model. **(A)** Results of neural network visualization. The positive weights are connected with red lines, and the negative weights are connected with gray lines. The thickness of the lines reflects the value of the weights. **(B)** A confusion matrix shows the classification ability of neural network in training dataset. **(C)** ROC curve shows the diagnostic ability of neural network in training dataset. **(D)** The expression levels of six hub DEARGs in training dataset. **(E)** A confusion matrix shows the classification ability of neural network in test dataset. **(F)** ROC curve shows the diagnostic ability of neural network in test dataset. **(G)** The expression levels of six hub DEARGs in test dataset.

Based on the above screened six diagnostic signatures, we used the GSE157628 dataset as the training set to construct the back propagation artificial neural network model using the R package “neuralnet”. After performing the preprocessing and scaling of this dataset, a neural network model with one hidden neuron layer was established. According to the output results of the neural network model (Figure 8A, Supplementary Table S3, and Supplementary Figure S4), the entire training was performed in 179 steps with an error of 0.021. Among the output results, the predicted weights of each hidden neuron layer were −14.758, −1.778, −5.728, and 13.268 for MMD. The confusion matrix and ROC curves show that the predicted scores (namely neuroMMD) present great diagnostic ability with AUC = 1 in the GSE157628 dataset (Figures 8B, C). The relative expression levels of these six diagnostic signatures are depicted through raincloud plots, indicating that *CD38*, *NOTCH11*, *TLR7*, and *ISG15* were upregulated, and *PTPN11* and *KAT2B* were downregulated (Figure 8D). We also recruited an external GSE141025 dataset that demonstrated the discriminatory performance of neuroMMD scores for MMD. The confusion matrix and ROC curves validated the great diagnostic ability of neuroMMD scores in MMD (Figures 8E, F). Although these genes from the verifying dataset showed the same expression trends as the training dataset, only CD38 and PTPN11 reached statistical significance (Figure 8G). Our RT-qPCR results also verified the same expression trends of CD38 and PTPN11 as the GSE157628 and GSE141025 datasets, and we also found the significant up-regulation of NOTCH1 of MMD patients in our results (Supplementary Figure S5).

### Identification of differential immune characteristics between moyamoya disease and controls

**FIGURE 9:**
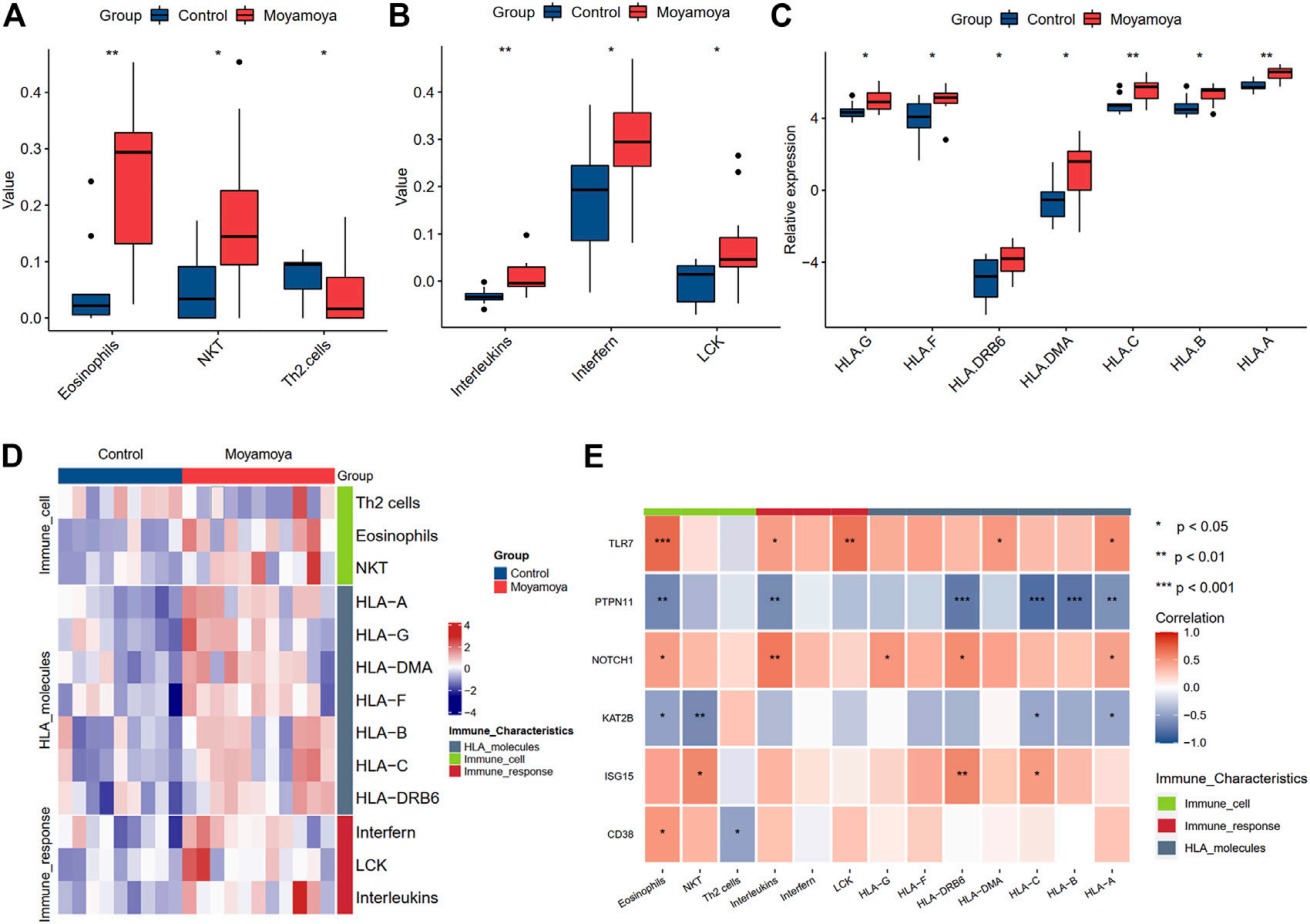
The significantly differential immune characteristics between MMD and controls. **(A)** The significantly differential immune cells. **(B)** The significantly differential immune activities. **(C)** The significantly differential expressed HLA molecules. **(D)** A heat map shows the landscapes of immune characteristics between MMD and controls. **(E)** The correlation of immune characteristics and six hub DEARGs. Significance level was denoted by *p‐value <0.05, **p‐value <0.01, ***p‐value <0.001.

Based on xCell analysis results, we identified three immune cells (eosinophils, natural killer T cells, and Th2 cells) with significant infiltration differences between diseased tissues and controls. Among them, eosinophils and NKT were increased in diseased tissues, while Th2 cells were decreased. We also assessed the differences in immune activities and responses between MMD and controls using the ssGSEA algorithm (Figure 9A). Three immune activities (interleukins, interferon, and LCK molecules) showed significant differences between groups (Figure 9B). Seven differential expressed HLA molecules were also discovered (Figure 9C), and all were upregulated. The differential immune characteristics between MMD and controls are depicted through a heatmap (Figure 9D).

The correlation analysis between six hub DEARGs and differential immune characteristics showed that the upregulated genes were positively correlated with increasing immune characteristics and negatively with decreasing immune characteristics. In contrast, the downregulated genes showed inverse effects.

### Establishment of miRNA-gene and drug-gene regulatory networks

**FIGURE 10:**
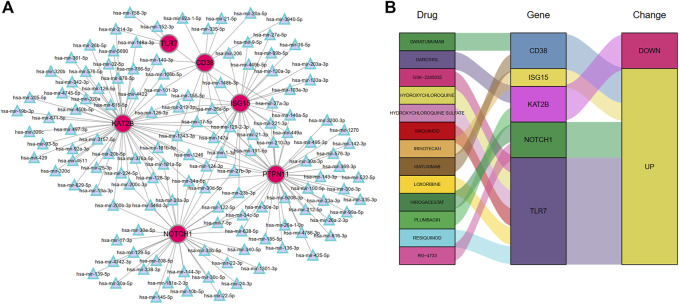
The miRNA-Genes regulatory network **(A)** and Drug-Genes interactions **(B)**.

To identify the regulatory and therapeutic mechanisms for MMD, we predicted the miRNA and drugs targeting the six hub DEARGs through the Tarbase and DGIdb databases, respectively. A total of 131 miRNAs were found to potentially regulate the hub ARDEGs (Figure 10A). Genes with the most regulated miRNAs were identified as *KAT2B* (regulated by 56 miRNAs), followed by *PTPN11* (43 miRNAs), *NOTCH1* (35 miRNAs), *ISG15* (29 miRNAs), *CD38* (13 miRNAs), and *TLR7* (5 miRNAs) (Supplementary Table S4). We also discovered that four miRNAs (mir-26a-5p, mir-1343-3p, mir-129-2-3p, and mir-124-3p) interacted with four hub genes.

The DGIdb was applied to predict possible medicines or molecular compounds reacting with the hub ARDEGs. After excluding drugs/compounds for which the interaction types with genes were not clear, 13 potential target drugs/compounds remained for MMD treatment (Figure 10B and Supplementary Table S5). Of these, six drugs interacted with *TLR7*; two targeted *CD38*, *KAT2B*, and *NOTCH1*; one drug targeted *ISG15*. *PTPN11* interacted with no potential drug.

## Discussion

As the most common pediatric cerebrovascular disease in Japan, the incidence of frequently recurrent ischemic episodes (transient ischemic attacks or strokes) is 70%–80% in children with MMD (Currie et al., 2011). MMD cannot be effectively treated with pharmacological interventions alone; therefore, surgical procedures for revascularization (direct, indirect, and combined bypass) are required. There is much controversy surrounding the optimal treatment for this disorder (Jang et al., 2017; Deng et al., 2018). The incidence of perioperative complications such as stroke, hyperperfusion syndrome, and acute thrombogenesis is also concerning (Kim et al., 2016). It is of profound significance to understand the pathophysiologic processes of MMD and prevent the occurrence of this disorder. However, the molecular etiology of MMD remains unclear. Previous studies have suggested the role of comorbidities as the link between autoimmune diseases and MMD (Kim et al., 2010; Chen et al., 2016). Therefore, analysis of ARGs may help determine the pathogenesis of MMD.

This study aimed to elucidate the critical processes and ARGs responsible for developing MMD by integrating bioinformatics and machine learning methods. Machine learning and artificial intelligence have become indispensable productivity tools for the 21st century for precision medicine. Machine learning and artificial intelligence differ from traditional biomedical research because they use huge volumes of data to uncover natural laws, which are then applied to medical research. The field of bioinformatics involves the development of computational tools and approaches for acquiring, storing, visualizing, and interpreting medical or biological data. Combining machine learning and bioinformatics will facilitate the generation, analysis, maintenance, and interpretation of information derived from molecular genetics tests. Apart from the field of oncology research, the integrated approach is widely applied in cardiovascular diseases, such as myocardial infarction (Wu et al., 2022), heart failure (Tian et al., 2020), and aortic valve calcification (Xiong et al., 2022). Through machine learning and bioinformatics technology, our study revealed the differential expression of ARGs and their potential biological functions in MMD for the first time. A predictive model with hub ARGs was constructed by using an artificial neural network. Immune cell infiltration, immune activities, and HLA molecule expression were investigated in MMD. The elucidated correlations between ARGs and immune characteristics may help further explain the interplay of ARGs and the immune microenvironment in MMD.

In our study, a total of 1696 DEGs (750 upregulated genes and 946 downregulated genes) between MMD and controls were screened. In biological function analysis, the pathways of autoimmune diseases (such as autoimmune thyroid disease, rheumatoid arthritis, the intestinal immune network for IgA production, and SLE), cell adhesion molecules (CAMs), and chylomicron remodeling were more enriched in MMD than controls. When overlapped with the ARGs, we further intersected 260 DEARGs involved in bacterial infectious disease, rheumatic disease, and collagen disease in DO analysis. Moyamoya vasculopathy in patients with the underlying causal condition is usually regarded as “MM syndrome”. MMD concurrent with Graves’ disease (GD) was first reported by Kushima et al. (1991). Over the past two decades, reports of these two concurrent diseases have increased (Tendler et al., 1997; Ni et al., 2014; Chen et al., 2015). The associations between thyroid function and thyroid autoantibodies with MMD were also discovered (Kim et al., 2010). A case-control study found that compared with control subjects, the thyroid function and thyroid autoantibodies are elevated in pediatric patients with MMD (Li et al., 2011), further supported by another study without age stratification (Lei et al., 2014). Studies about SLE associated with MM syndrome have rarely been conducted and are published mostly in the form of case reports (El Ramahi and Al Rayes, 2000; Jeong et al., 2008). According to a recent review, MMD complicated with SLE mostly occurred in female patients [84.6% (11/13)], and most of these patients developed MMD by the age of 30 years (Tanaka et al., 2020). Among the 13 patients, 10 were from East Asian countries. Complications with rheumatoid arthritis and MMD are rare (Paciaroni et al., 2005). The common molecular characteristic of SLE and rheumatoid arthritis is the change in CAMs (da Rosa Franchi Santos et al., 2020), which was also identified by our enrichment analysis in MMD. CAMs, which are transmembrane proteins that facilitate cell-to-cell or cell-to-extracellular matrix binding, may be categorized into three different types named immunoglobulin supergene family members, selectins, and integrins. CAMs regulate the inflammatory response and endothelial function. Therefore, they may be targeted in cardiovascular disease (Kunutsor et al., 2017), such as atherosclerosis (Ling et al., 2012) and ischemic stroke (Yilmaz and Granger, 2008). DIAPH1 may be a novel MMD risk gene that impairs vascular cell actin remodeling that may cause neointimal expansion and progressive narrowing of the bilateral internal carotid arteries in MMD pathogenesis (Kundishora et al., 2021). Soriano et al. (2002). found significantly elevated levels of soluble CAMs in the cerebrospinal fluid of children with MM syndrome compared with the control group, suggesting the potential roles of CAMs in MMD. These results indicate the crucial association between MMD and autoimmune diseases. By examining the common pathogenesis of these disorders, we can clarify the etiology of MMD. CAMs may act as a bridge that triggers the common pathogenesis processes.

We also performed enrichment analysis of DEARGs in MMD, suggesting that infectious diseases (such as *Staphylococcus aureus* infection, Epstein-Barr virus infection, and *tuberculosis*), cAMP signaling pathway, and PI3K-Akt signaling pathway were involved. The infection hypothesis has occasionally been proposed as one of three mechanisms while investigating the pathogenesis of MMD, apart from autoimmune and HLA abnormality. Infections associated with MMD have been reported in many cases, including bacterial meningitis due to pneumococcus, tuberous infection, and viral infection by the varicella-zoster virus, Epstein-Barr virus, and *Leptospira* infection (Houkin et al., 2012). Czartoski proposed that the inflammation and subsequent post-infectious autoimmune response associated with meningitis can lead to a progressive vasculopathy, which may cause arterial occlusions in MM syndrome after autopsy in a patient with pneumococcal meningitis (Czartoski et al., 2005). Despite suggesting a possible infectious cause in MMD, these results were only based on case studies, and no specific pathogen has been identified. A large-sample study is indispensable to finding the relationship between infections and MMD. The DEARGs we identified as associated with these diseases may provide a molecular-level explanation.

A PPI network was constructed based on the DEARGs to explore relationships among proteins. We found 15 potential genes overlapping the top 30 genes identified by six algorithms (Closeness, Degree, MCC, MNC, and Radiality) in cytoHubba. To further screen out the hub genes, three machine learning methods (LASSO regression, RF, and SVM-RFE) were applied, and six genes (*CD38*, *PTPN11*, *NOTCH1*, *TLR7*, *KAT2B*, and *ISG15*) were selected to construct a predictive model using BP artificial neural network in the GSE157628 dataset. The GSE141025 dataset also verified the predictive performance with great diagnostic ability, which proved the applicability of our model. Type I interferons (IFNs) induce the expression of over 500 genes, collectively referred to as IFN-stimulated genes (ISGs). ISG15 is a ubiquitin-like protein that can conjugate to substrate proteins (ISGylation) in response to microbial infection. This IFN-α/β-inducible ISG15 does not serve as a substrate for ISGylation-based antiviral immunity but for regulating IFN-α/β by USP18 and preventing IFN-α/β-dependent auto-inflammation (Zhang et al., 2015). The antiviral and antineoplastic roles of ISG15 have been extensively studied (Mustachio et al., 2018; Perng and Lenschow, 2018). RNF213 is an interferon-induced mega protein frequently mutated in MMD as a susceptibility gene (Liu et al., 2011). A recent study pointed out that RNF213, an ISG15 interactor, can act as a sensor for ISGylated proteins to counteract infection (Thery et al., 2021). In our immune infiltration analysis, we observed that the activity of IFNs and the expression of ISG15 genes were higher in MMD than in controls. Therefore, the overexpression of ISG15 induced by IFNs may be involved in the pathogenesis of MMD through its interaction with RNF213. This finding may provide a new direction for basic experiments in the future. *PTPN11*, the gene encoding the protein tyrosine phosphatase SHP2, is a ubiquitously expressed non-receptor tyrosine phosphatase that regulates cell survival, proliferation, differentiation, migration, and adhesion. Germline mutations in *PTPN11* cause Noonan syndrome, the clinically related LEOPARD syndrome (LS), and leukemogenesis (Tartaglia et al., 2006; Alfayez et al., 2021). Seki et al. found that the expression of SHP2 was markedly elevated in the thickened aortic intima in rats with balloon-induced injury in an atherosclerosis animal model (Seki et al., 2002). The inhibition of SHP2 can protect against atherosclerosis by inhibiting smooth muscle cell proliferation (Chen et al., 2018). The most prominent pathological change in MMD is the inner elastic lamellar’s breakage and smooth muscle cells’ destruction and proliferation in the tunica media (Huang et al., 2017). There are some common pathogenesis links between MMD and atherosclerosis. MMD susceptibility variant RNF213 p. R4810K can increase the risk of recurrent cerebrovascular events, such as ischemic stroke caused by large-artery atherosclerosis (Okazaki et al., 2019; Kim et al., 2021). Considering no direct evidence connecting *CD38*, *PTPN11*, *NOTCH1*, *TLR7*, and *KAT2B* with the pathogenesis of MMD, their roles in atherosclerosis may also provide a new perspective and direction for future research on the molecular targeted therapy of MMD (Salagianni et al., 2012; Briot et al., 2015; Xu et al., 2016; Qi et al., 2021).

Considering the important roles of immune activities in MMD, we also studied the immune characteristics from the perspective of immune cell infiltration, activities of immune responses, and HLA molecule expression. Our results showed that the abundance of eosinophils and natural killer T (NKT) cells is significantly elevated while Th2 cells were decreased in MMD compared to controls. The expression levels of HLA-A, HLA-B, HLA-C, HLA-DMA, HLA-DRB6, HLA-F, and HLA-G were significantly upregulated in MMD. The abnormality of HLA is considered one of the molecular mechanisms leading to the occurrence of MMD. Hong et al. (2009) found that the phenotype frequencies of HLA-DRB1(*) 1302 and DQB1(*) 0609 were significantly increased in familial MMD compared to both controls and non-familial Korean patients with MMD. In a Japanese case-control study on MMD, the HLA-DRB1*04:10 allele was found to be a predisposing genetic factor, and the frequency of autoimmune thyroid diseases was increased in HLA-DRB1*04:10-positive patients with MMD compared with that in HLA-DRB1*04:10-negative patients with MMD (Tashiro et al., 2019). Recent research in Chinese Han population indicated that the genetic polymorphism of HLA-DQA2 and HLA-B was identified as a risk factor for MMD (Wan et al., 2021).

Gene-miRNAs modify the appearance of proteins with the progression of diseases by targeting their main targets. In this study, we also constructed a gene-miRNA regulatory network, and four miRNAs (mir-26a-5p, mir-1343-3p, mir-129-2-3p, and mir-124-3p) were identified because of their interaction at least with four hub DEARGs. Mir-26a-5p can alleviate cardiac hypertrophy and dysfunction (Shi et al., 2021) and protect against myocardial ischemia/reperfusion injury (Xing et al., 2020). Mir-1343-3p plays a significant role in the development of human cancers such as lung cancer (Zhang et al., 2020), colorectal carcinoma (Bhat et al., 2022), and hepatocellular carcinoma (Mou and Sun, 2022). The involvement of mir-1343-3p in cardiovascular diseases is also reported (Sharma et al., 2020). The potential role of mir-129-2-3p in ischemic stroke was identified by suppressing *SYK* gene expression (Huang et al., 2019). Mir-124-3p contributes to the development of different cardiovascular diseases, such as atherosclerosis, myocardial infarction, and ischemic stroke (de Ronde et al., 2017; Badacz et al., 2021). These four miRNAs may be used as interventional targets for examining the mechanisms of ARGs in MMD since they interact with at least four hub DEARGs. Moreover, a total of 13 potential drugs/compounds were predicted for MMD treatment by targeting the hub ARGs in our study.

Further studies are warranted to address some limitations of the present study. First, although the diagnostic model constructed by an artificial neural network performed well in the training and testing datasets, the sample size was very small, especially for the validation dataset, where only four samples were available in each group. Therefore, studies with larger sample sizes are essential. Second, in the GSE157628 dataset, six control samples were collected from the MCA of patients with ICA aneurysms. Considering the different hemodynamic and genetic effects, the normal artery of patients with aneurysms may differ from normal vessels at the transcriptional level. However, the collection of normal vessels from healthy control, in essence, is against medical ethics. Setting the normal artery from patients with aneurysms as the control group in studying MMD is acceptable (Kanamori et al., 2021). Third, the results were based on bioinformatics and conducted RT-qPCR, but *in vitro* and *in vivo* experiments should be conducted to verify the results.

## Conclusion

In our analysis, a total of 260 DEARGs were identified in MMD, which were involved in autoimmune-related diseases and immune responses. Six ARGs (*CD38*, *PTPN11*, *NOTCH1*, *TLR7*, *KAT2B*, and *ISG15*) were selected by three machine learning methods (LASSO regression, RF, and SVM-RFE). They were finally used to construct a predictive model using BP artificial neural network that could be used to identify patients with MMD. Immune infiltration analysis showed that immune activities and HLA expression levels in MMD were enhanced. Finally, a gene-miRNA network was prepared, and pharmacological agents targeting hub genes were predicted as potentially effective in treating MMD.

## Data Availability

The original contributions presented in the study are included in the article/Supplementary Material, further inquiries can be directed to the corresponding author.
