# Supplementary material for: Comprehensive molecular analyses of an autoimmune-related gene predictive model and immune infiltrations using machine learning methods in moyamoya disease
Source: Front Mol Biosci. 2022 Dec 20;9:991425. doi: 10.3389/fmolb.2022.991425 (PMC9808060; doi:10.3389/fmolb.2022.991425)
Supplement: Supplementary file 1 [file DataSheet1.docx]

Supplementary Materials


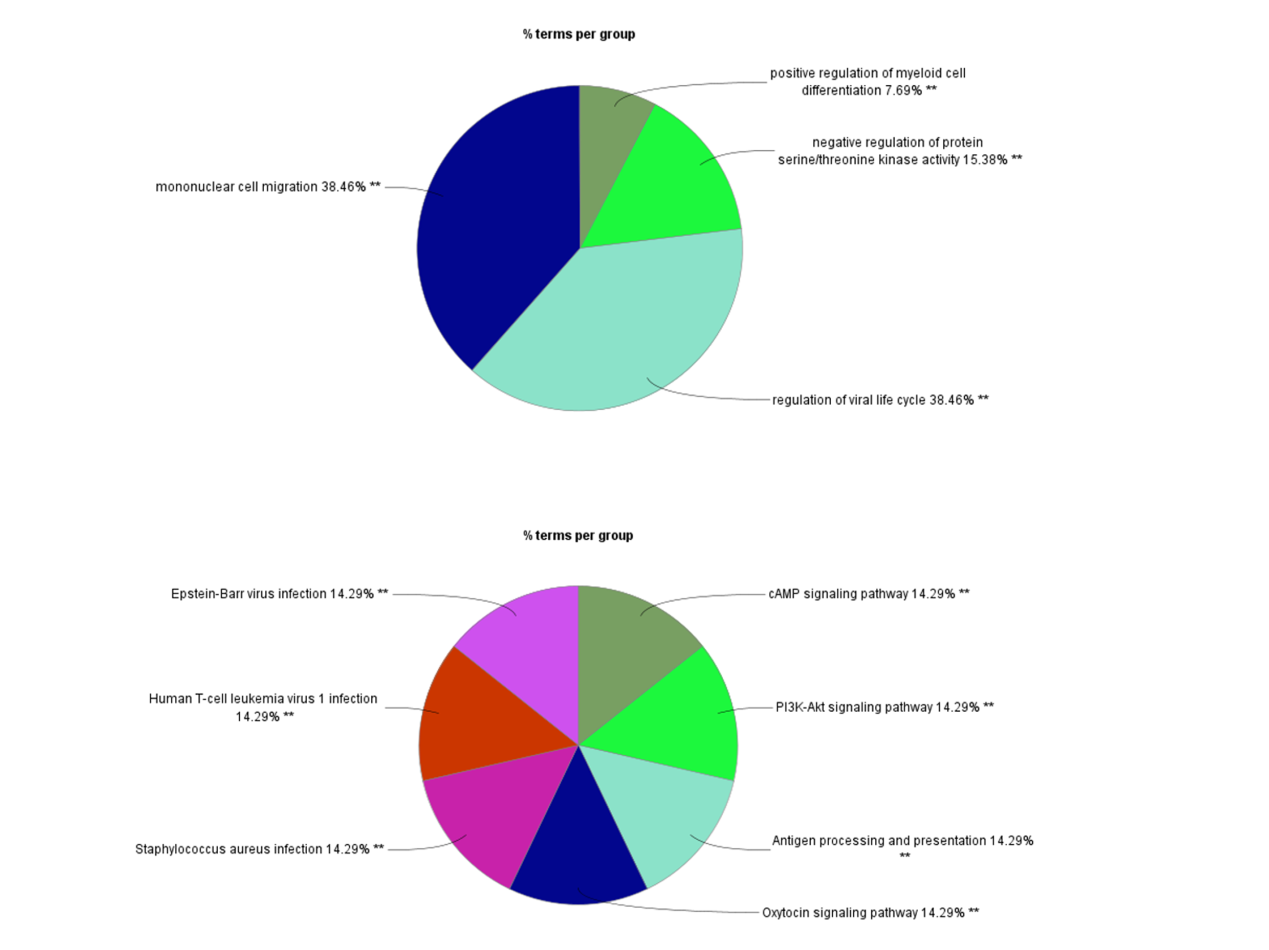


Figure S1. The pie charts for the BP and KEGG enrichments of DEARGs.


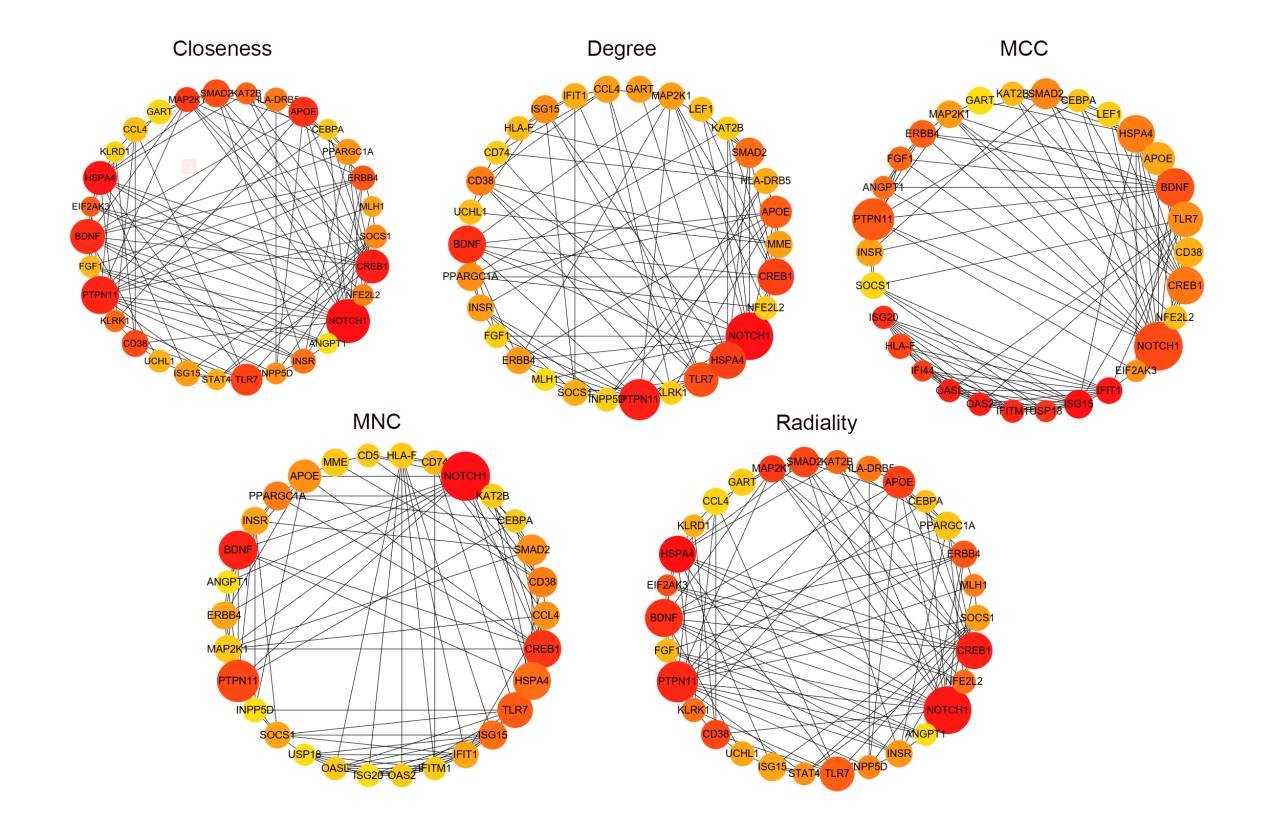


Figure S2. The top 30 genes identified by five methods in cytoHubba.


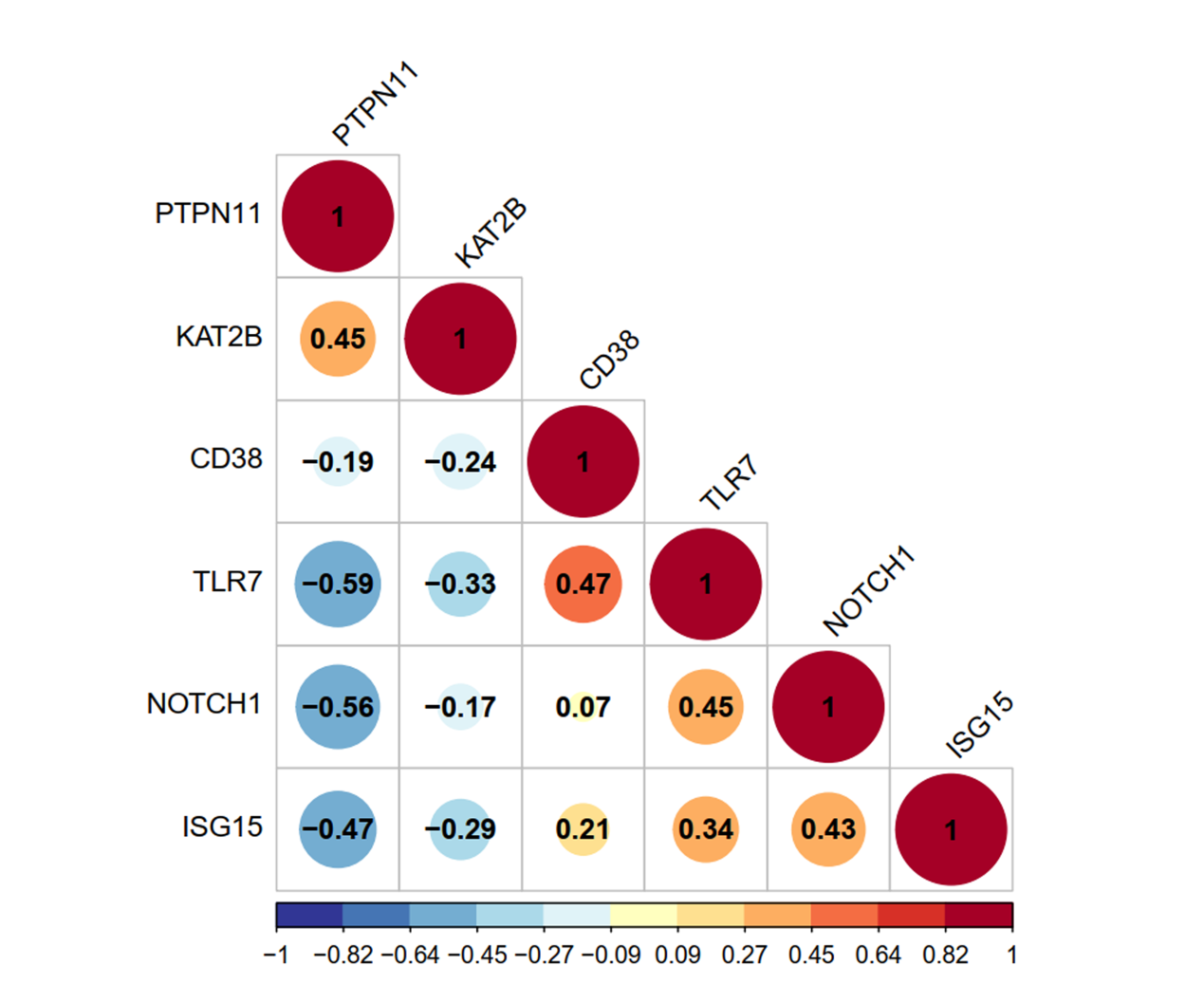


Figure S3. The correlations of six hub DEARGs.


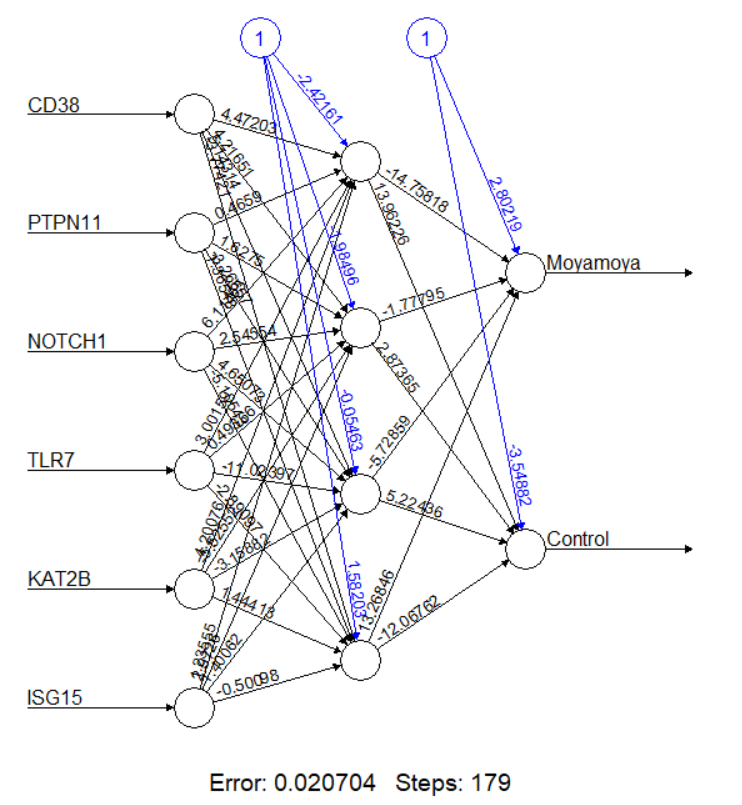


Figure S4. Visualization of back propagation artificial neural network result.


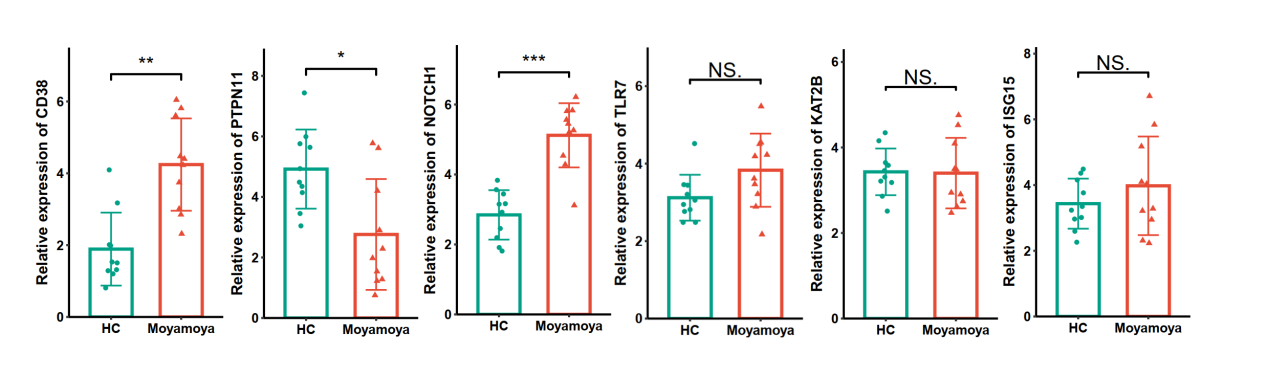


Figure S5. RT-qPCR verifying the expression levels of six hub genes in our dataset.
